# Providing a comprehensive approach to oil well blowout risk assessment

**DOI:** 10.1371/journal.pone.0296086

**Published:** 2023-12-20

**Authors:** Mostafa Satiarvand, Neda Orak, Katayoon Varshosaz, Elham Mobarak Hassan, Mahboobeh Cheraghi

**Affiliations:** Department of Environment, Ahvaz Branch, Islamic Azad University, Ahvaz, Iran; Istanbul University: Istanbul Universitesi, TURKEY

## Abstract

Drilling is one of the most dangerous activities in the oil and gas process industries. Therefore, a holistic approach was presented to prevent and control risks and reduce the uncertainty of blowouts, kick scenarios, and control layers based on the Fuzzy Bayesian Network (FBN). Four independent protection layers (IPLs) were used to evaluate the blowout outcome, and the failure probabilities of IPL1 and IPL2 were calculated with Fault Tree Analysis (FTA). Then, different events were transferred to the Bayesian Network (BN) in GeNIe software, and updated the probabilities. The Fuzzy Fault Tree (FFT) results reveal that the failure probabilities for IPL1 and IPL2 amount to 8.94×10^−4^ and 4.97×10^−21^, respectively. Also, the blowout probability results based on FFT were larger than FBN. According to FBN, the probability of the kick event was equal to 6.60×10^−34^. Sensitivity analysis showed that X1 (Mud volume/flow change) had the highest degree of importance in the blowout of oil wells. The results of this study can be used in both a preventive and reactive approach. Deductive and inductive reasoning, the dynamic nature and conditional dependencies, and causal relationships between events can make the model more realistic.

## 1. Introduction

Owing to the diverse array and substantial quantities of hazardous and flammable chemicals, process industries are deemed high-risk sectors that are susceptible to catastrophic accidents [1, 2]. Drilling is one of the process industries that ranks among the most critical operational sectors worldwide with significant risks due to its substantial volumes of hydrocarbon and chemical materials. Furthermore, its dense workforce and equipment combined with limited time for emergency responses exacerbate these risks [3]. The harsh work environment has also had a significant effect on occupational hazards in this industry. Compared to other oil fields, oil and gas drilling operations have the highest rate of accidents and the highest number of lost work days [4, 5].

### 1.1 Kick

Exploration and development of oil and gas industries are always associated with many risks such as human losses, environmental pollution, and loss of equipment and resources. A catastrophic occurrence that poses great significance and substantial financial repercussions within the drilling sector is known as a well blowout. This perilous event has the potential to inflict harm upon numerous individuals, ignite fierce fires, trigger massive explosions, contaminate the surrounding ecosystem with millions of barrels of oil, and consequently give rise to severe environmental catastrophes [6, 7].

Blowout is one of the rare and critical events that often cause heavy damage and are difficult to control [6]. One of the most important and costly accidents in the drilling industry is a well blowout, which can cause injuries to a large number of personnel, fires, and large explosions, spill millions of barrels of oil into the surrounding environment, and cause environmental disasters [8]. Blowout is the uncontrolled flow of hydrocarbons (gas, oil, and gas condensate) or salt water from the well to the surrounding environment, which occurs as a result of an uncontrolled kick [9]. Kick is also the unwanted intrusion of formation fluids into the well due to a lack of Loss of Well Control (LWC). In other words, in this case, the pore pressure is more than the pressure exerted by the drilling fluid column at the bottom of the well, or Bottom Hole Pressure (BHP) [9, 10].

While engaged in drilling, if the pressure of the geological structure exceeds that exerted by the fluid column within the well, it allows for the ingress of the formation liquid, which may manifest as water, oil, or gas. This incoming fluid reaches the surface after some time, and if the necessary measures are not taken to control the well, it may cause a great loss of life and money, especially if the incoming fluid is gas, and enters the well. It is more dangerous because the gas reaches the surface quickly due to its lightness, and the closer it gets to the surface, the gas volume increases due to the decrease in the hydrostatic pressure of the drilling fluid [9].

If a Kick is not detected and properly controlled in time, the phenomenon of a well blowout is not far from the mind. For this reason, the control of seepage plays a vital role in the prevention of blowouts [11]. Therefore, it is necessary to apply several safety barriers to prevent blowouts.

### 1.2 Control barriers

Following ISO 13702, prevention entails a deliberate decrease in the probability of a perilous incident transpiring. An additional aspect brought forth by this standard is the introduction of "control" and "reduction" as techniques aimed at limiting both the escalation and impact resulting from such hazardous occurrences [12]. Hollnagel simplifies the purpose of safety barriers to two fundamental functions: prevention and protection. These preventive measures are contemplated before a particular event, serving as a means to defend against its onset. Once an initiating event has taken place, these precautionary barriers shift their function towards safeguarding against further harm [13].

Therefore, in this study, to prevent blowouts, the factors that cause them should be prevented. It should be noted that some kicks and blowouts are unavoidable, but concerted efforts are needed to minimize them [9]. Well-control operations include technical, managerial, and organizational measures that are carried out through the prevention of kicks, their timely detection, blowout prevention, and well-kill operations [14, 15]. Risk analysis is one of the most important tools for assessing risks, designing risk reduction measures, and increasing the level of safety in process industries [1, 16].

### 1.3 Limitations of conventional risk assessment

Therefore, the failure to assess and identify comprehensive health and environmental safety risks can cause catastrophic consequences. The use of integrated, reliable, and dynamic methods is very effective in identifying potential hazards, causes, and consequences and considering control measures to mitigate risks. Conventional methods of risk assessment due to operational variables such as pressure, temperature, and flow which are constantly changing cannot be effective in identifying blowouts and preventing the consequences of blowouts and require the use of accurate and up-to-date methods [17]. Most risk assessment studies have had several limitations and uncertainties. In these studies, an attempt has been made to reduce one or more limited uncertainties. But in this study, the combined and comprehensive approach of Bow-Tie and fuzzy Bayesian method was used, which provides a dynamic structure for quantitative risk analysis (QRA) in addition to reducing uncertainty.

Fuzzy sets are employed in situations of uncertainty to advocate for a multi-valued logic rather than the conventional two-valued logic. Consequently, fuzzy logic presents itself as a favorable approach to risk management that addresses both unpredictability and qualitative variables [18, 19]. Numerous distinct and unchanging methodologies have been devised for risk assessment, including event tree analysis (ETA), fault tree analysis (FTA), and Bow-Tie analysis (BTA). However, these conventional techniques prove inadequate when evaluating intricate systems, particularly if said systems consist of supplementary components or display dynamic behavior with time-dependent parameters [20]. Currently, there is an expanding employment of Bayesian networks (BN) to oversee the assessment and control of risks in process facilities.

In the Bayesian method, incident precursor data is used as a probability function from the Bayes theorem to update prior beliefs about event probability [21–24]. BN is a method renowned for its versatility through visual representation coupled with robust logic-driven decision-making processes regarding process equipment safety evaluations [25]. Several studies have been done using different tools to assess the risk of process industries and different tools have been used to calculate blowout probabilities. The studies of Cai et al. [26, 27], Mutlu et al. [28], Meng et al. [29, 30], Chang et al. [31], and Liu et al. [5] are examples of cases where BN and dynamic Bayesian network (DBN) were used for blowout risk assessment. Di Maio and colleagues also used the Dynamic Event Tree (DET) combined approach with BN to analyze the blowout accident in an oil deep-water well [32]. Bhandari et al.’s study also used BN to perform a dynamic safety analysis of Managed Pressure Drilling Operations (MPD) and Underbalanced Drilling (UBD) operations in deep water. The results showed that UBD has a higher probability of kick and blowout compared to MPD technology [33]. Each of these studies has many strengths and weaknesses and they have tried to use methods for analysis according to the prevailing conditions. Abimbola et al also used Bow-Tie models to map safety challenges and operating pressure regimes in constant bottom-hole pressure drilling technique [34].

However, guided by the preceding discourse, this study adopts an all-encompassing strategy pursuing preventive and reactive analysis about Kick and blowout assessments alongside barrier analysis practices. To accomplish these objectives effectively fault tree analyses and leveraging fuzzy logic principles shall be entwined with BN methods throughout this research endeavor.

There is a possibility of safety, health, and environmental consequences in the Yaran oil field in HorolAzim, which is the habitat of aquatic animals and birds. The basic reasons for conducting this study include the difficulty of quick access to the oil pads located on Hour for emergency measures, the commonality of the Yaran field with Iraq and the damage caused by operational interruptions due to incidents caused by well blowout, and failure to conduct comprehensive and quantitative risk assessment studies with new approaches to reduce uncertainties. Therefore, the present study was designed to reduce the limitations to analyze the root causes of the blowout of exploratory and production wells in the Yaran oil field.

## 2. Material and methods

**Fig 1:**
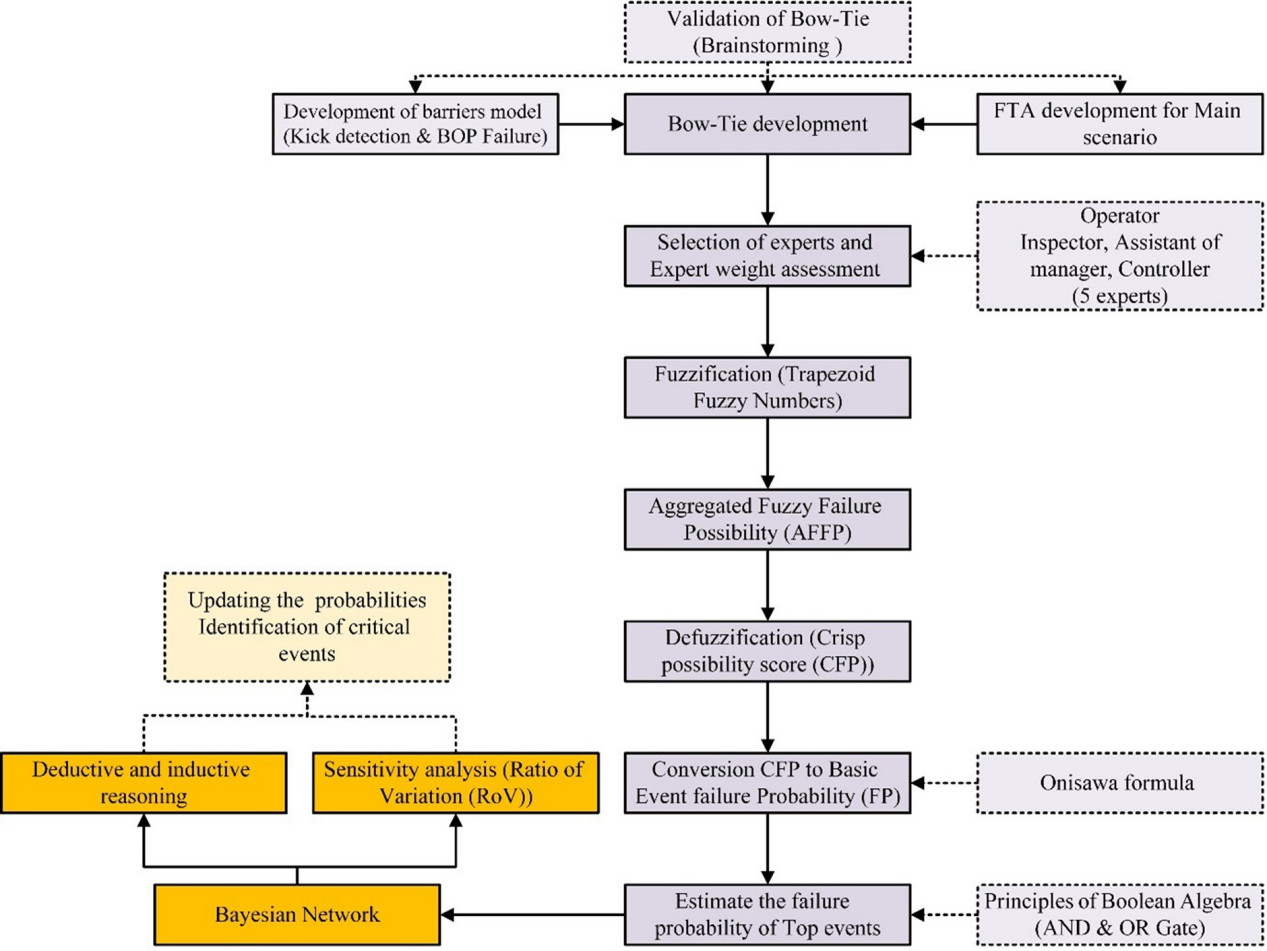
Study flowchart.

The present study was conducted on the oil wells of the Yaran field, 130 kilometers southwest of Ahvaz, at the border zero point in the HorolAzim region during the drilling operation. Fig 1 shows the overall flowchart of the study. According to Fig 1, the first step in this study was the development of the Bow-Tie model and error trees related to the two control layers of the Kick detection and BOP System. The probabilities of the other two layers were obtained from the databases. Experts’ opinions and brainstorming techniques were used to validate the structures (Bow-Tie Development stage in Fig 1). In addition, the period for conducting this study is from 2022 to 2023.

### 2.1 Fuzzy logic

In this specific analysis, the implementation of fuzzy logic and expert insights were utilized to assess the likelihoods associated with key occurrences within fault trees. These trees pertain to the primary scenario known as "Kick," as well as the barriers involved in Kick detection and the Blowout Preventer (BOP) system. The information related to IPLs 3 and 4 was derived from Khakzad et al.’s investigation due to their resemblance to our study [14]. The study consisted of a 5-person expert panel as can be seen in Table 1 and Fig 1. The experts of the study were people who had the necessary knowledge about the drilling and oil wells processes and were familiar with the structure of the Bow-Tie method. It must be noted that experts possess distinct specializations and varied work experience, leading them to hold diverse perceptions regarding these events. Thus, a weighting factor (WF) was taken into account. The WF for each expert was determined by summing up their respective Likert ratings, which were then divided by the cumulative total of points obtained by all participating experts [35]. To collect these scores from each expert, collaborative assessments referred to Table 1 as a benchmark. Each item under consideration garnered input from different experts until reaching a final consensus represented numerically. Eq 1 proved invaluable to accomplish this task effectively [36]. (Expert Weight Assessment step in Fig 1).


$$M_{i}=\sum_{i=1}^{m}W_{j}A_{ij},j=1,2,\ldots .,n$$ (1)

**Table 1:** Expert weighting.

Row	Title	Description	Score
1	Job Title	Manager & Deputy	4
		Inspector، Assistant of manager، Controller	3
		Site supervisor ، Foreman	2
		Operator	1
2	Experience	30	4
		20–30	3
		10–20	2
		5–10	1
3	Education	PhD	5
		Bachelor, Master	4
		Diploma	3
		Holder of an occupational diploma	2
		High school dropout	1
4	Age	<50	4
		50–40	3
		40–30	2
		30>	1

The "fuzzy fault probability" (Mi) is determined by summing the fuzzy values of event I, while the linguistic variable assigned to event i by expert j is represented as Aij. Also, m denotes the total number of events, and n indicates the total number of experts, the weighting score of expert J is denoted as Wj.

The process for weighting the experts was carried out using Table 2 and Eq 6.

**Table 2:** Fuzzy scales.

Linguistic terms	Trapezoid Fuzzy Numbers			
Very Low (VL)	0.2	0.1	0	0
Low(L)	0.4	0.25	0.25	0.1
Medium(M)	0.7	0.5	0.5	0.3
High(H)	0.9	0.75	0.75	0.6
Very High (VH)	1	1	0.9	0.8

The phase of fuzzification and subjective evaluation of experts produces a set of qualitative data that represents the probability of failure of the main event. Then, to calculate the probability of occurrence of basic events, linguistic variables expressing experts’ opinions were defined as a failure probability distribution (FPD). In recent studies, trapezoidal and triangular fuzzy numbers have been known to be more useful for risk assessment [37–39]. Saaty and Ozdemir suggested numbers between 5 and 9 for expert judgment [40]. In the present study, 5 language terms were used (Table 2). If the number of linguistic terms is large, it may confuse the expert in answering the questions.

Then the consensus of the experts’ opinions was carried out as the aggregated fuzzy failure possibility (AFFP). Subsequently, the opinions of the experts were combined into a single opinion according to the identified basic events. Then defuzzification or determining the crisp failure possibility (CFP) was done using the center of the area (CoA) method. Therefore, the AFFP values resulting from the consensus stage should be converted into a definite number [16, 41]. Eqs 2 and 3 are related to the consensus of opinions and defuzzification. Then the conversion of CFP to the probability of failure (FP) was done using Onisawa’s relation (Eqs 4 and 5) [42]. In this relation, K is an intermediate variable that is only a function of CFP.

$${\text{X}}^{*}=\frac{\int \mu_{i}(x)xdx}{\int \mu_{i}(x)}$$ (2)

$$\begin{array}{l} \mu_{∼A}(x)=\left\{\begin{array}{c} \frac{x-a_{1}}{a_{2-a_{1}}},a_{1\leq x\leq a_{2}}\\ 1,a_{2}\leq x\leq a_{3}\\ \frac{a_{4-x}}{a_{4-a_{3}}},a_{3}\leq x\leq a_{4}\\ 0,x>a_{4} \end{array}\;X^{*}=\frac{\int_{a_{1}}^{a_{2}}\frac{x-a}{a_{2}-a_{1}}xdx+\int_{a_{2}}^{a_{3}}xdx+\int_{a_{3}}^{a_{4}}\frac{a_{4}-x}{a_{4}-a_{3}}xdx}{\int_{a_{1}}^{a_{2}}\frac{x-a_{1}}{a_{2}-a_{1}}dx+\int_{a_{2}}^{a_{3}}dx+\int_{a_{3}}^{a_{4}}\frac{a_{4}-x}{a_{4}-a_{3}}dx}\right.\\ =\frac{1}{3}\times \frac{{\left(a_{4}+a_{3}\right)}^{2}-a_{4}a_{3}-{\left(a_{1}+a_{2}\right)}^{2}+a_{1}a_{2}}{\left(a_{4}+a_{3}-a_{1}-a_{2}\right)} \end{array}$$ (3)

$$FP=\left\{\begin{array}{c} \frac{1}{10^{K}},FPS\neq 0\\ 0,FPS=0 \end{array}\right.$$ (4)

$$K=\left[{\left(\frac{1-FPS}{FPS}\right)}^{1/3}\right]\times 2.301$$ (5)

Then, the probability of occurrence of top and intermediate events was estimated using Eqs 6–8 according to the type of the corresponding gate (OR and AND gate), which are obtained according to the principles of Boolean algebra [43].


$$P_{OR}=1-\Pi_{i=1}^{n}\left(1-P_{i}\right)$$ (6)

$$P_{AND}=\Pi_{i=1}^{n}P_{i}$$ (7)

$$P_{Te}=\Pi_{j\in M}\left(1-\Pi_{BE_{i\in Qj}}\left(1-P_{i}\right)\right)$$ (8)

Where Pi = the probability of occurrence of the basic event (BEi); Qj = basic event or a group of basic events.

Then, the probability of the kick result was also obtained from Eq 9 according to the failure of the barriers.


$$P_{r}(c)=P_{r}(TE)\times \Pi_{j=1}^{n}P_{r}(E)$$ (9)

Where Pr (c) = the probability of each outcome; Pr (Te) = the probability of the top event; Pr(E) = the probability of failure or work of independent protection layers (IPLs).

### 2.2 Bayesian network

The FBN has been widely acknowledged as a reliable methodology for assessing safety across various industrial and process incidents [1, 14, 16, 44]. The FBN can leverage Bayes theory as a means to continually reassess the likelihood of fundamental events transpiring based on the acquisition of fresh evidence, such as incident occurrence statistics, real-time process monitoring data, and near-misses. This enables the calculation of updated probabilities [44, 45].

Within this scientific investigation, the events were categorized into three groups: basic, intermediate, and top. These events were meticulously documented within the academic version of GeNIe software; the root node representing basic events, the middle node symbolizing intermediate events, and the central node embodying top events. Moreover, Conditional Probability Tables (CPTs) were formulated for various nodes and subsequently updated to assess their respective probabilities [46, 47].

Also, inductive reasoning with Eq 10 was done using Bayesian capability in GeNIe software. Thus, the probabilities of intermediate and top events were calculated according to the gate type bottom-up (from basic event to top event). Eq 10 is used in the FBN of the joint probability distribution of variables.


$$P(U)=\Pi_{i=1}^{n}P\left(X_{i}|Pa\left(X_{i}\right)\right)$$ (10)

Where P(U) = the joint probability distribution; Pa(Xi) = is the parent set of the Xi.

Eq 11 is also used to update the prior probabilities of events according to evidence (E) [1].


$$P(U|E)=\frac{P(U).P(E|U)}{\sum_{U}P(U).P(E|U)}=\frac{P(U,E)}{\sum_{U}P(U,E)}$$ (11)

Then the CPTs of different nodes were completed based on relevant gates according to relations 12 and 13. Y1 and Y2 can be root nodes and Z1 and Z2 can be intermediate nodes or central nodes [17].

For an OR gate:

$$\begin{array}{c} \text{P}(\text{Z1}=1|\text{Y1}=0,\text{Y2}=0)=0\\ \text{P}(\text{Z1}=1|\text{Y1}=1,\text{Y2}=0)=1\\ \text{P}(\text{Z1}=1|\text{Y1}=0,\text{Y2}=1)=1\\ \text{P}(\text{Z1}=1|\text{Y1}=1,\text{Y2}=1)=1 \end{array}$$ (12)

For an AND gate:

$$\begin{array}{c} \text{P}(\text{Z2}=1|\text{Y3}=0,\text{Y4}=0)=0\\ \text{P}(\text{Z2}=1|\text{Y3}=1,\text{Y4}=0)=0\\ \text{P}(\text{Z2}=1|\text{Y3}=0,\text{Y4}=1)=0\\ \text{P}(\text{Z2}=1|\text{Y3}=1,\text{Y4}=1)=1 \end{array}$$ (13)

One notable attribute of BN is its capacity for deductive reasoning. Therefore, the probability of the central node Z was calculated as P(accident|event) using Eq 14 for predictive analysis, while the probability of root node Yi in the form of P(event|accident) was calculated using Eq 15 for diagnostic analysis [17].


$$P(Z=1)=\sum_{\lambda (Z)}P(Z=1|\lambda (Z))P(\lambda (Z))=\sum_{\lambda (Z)}P(\lambda (Z),Z=1)$$ (14)

$$P\left(X_{i}=1|Z=1\right)=\frac{P\left(Z=1|X_{i}=1\right)}{P(Z=1)}$$ (15)

Furthermore, within this study’s context, FBN played a pivotal role in identifying the most critical events. Henceforth following such identification via FBN methodology, sensitivity analysis was conducted aiming to discern influential root nodes responsible for system failures across different processes due to their varying significance.

## 3. Results

**Fig 2:**
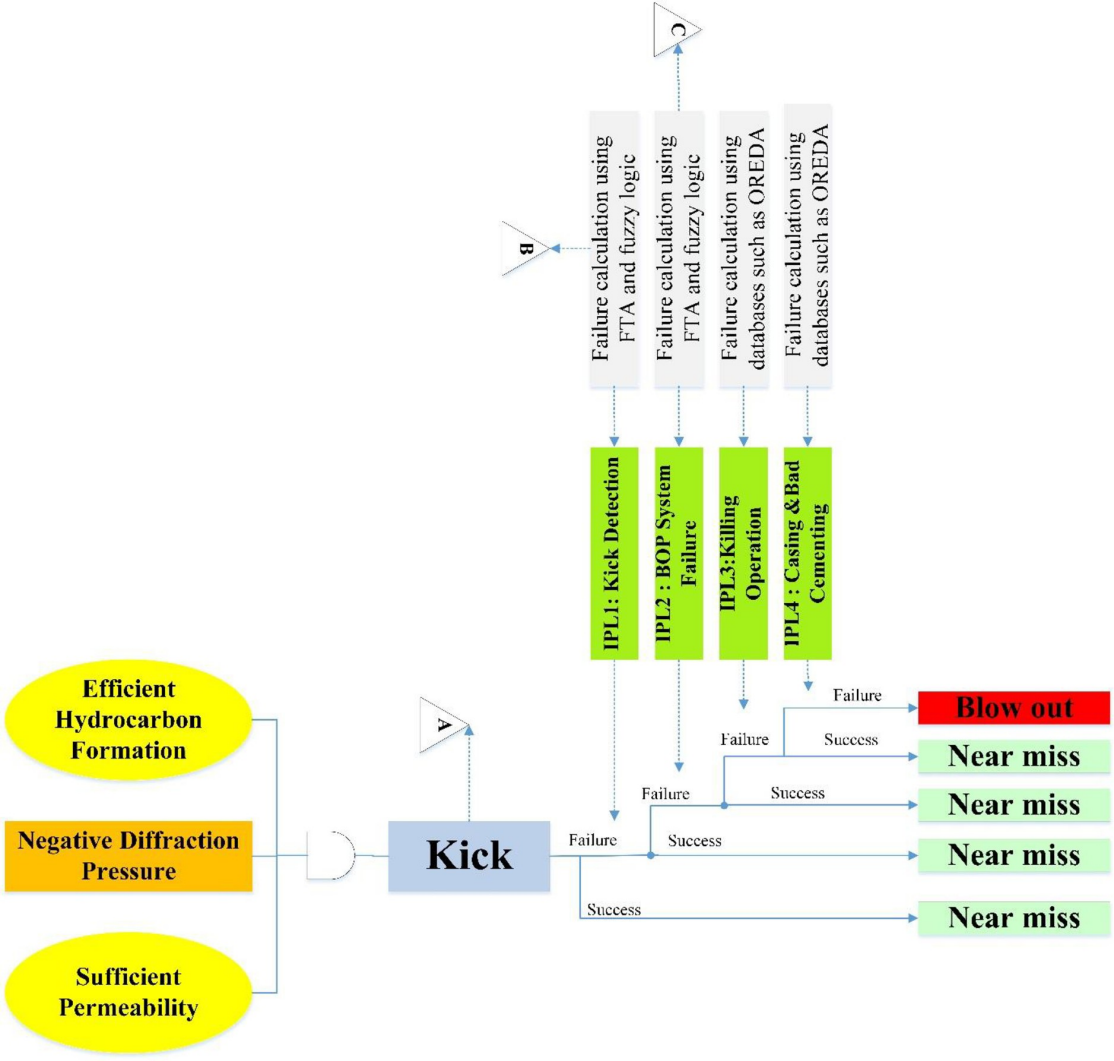
Static Bow-Tie model.

Initially, a panel of industry experts was assembled and the findings related to the fault tree and events were verified with expert opinions and necessary modifications. Fig 2 shows the overview of the study; in this part, we describe the findings in detail. According to Table 3, experts were weighted, and experts 4 and 5 had the highest weighted average (0.18).

**Table 3:** Expert weighting to estimate the probabilities of basic events.

Expert	Tile	Experience	Education	Age	Weight Index	Expert’s Weight score	Rank
1	Inspector Assistant Manager, Controller	20–30	Bachelor, Master	40–50	13	0.024	3
2	Inspector Assistant Manager, Controller	10–20	Bachelor, Master	30–40	12	0.022	4
3	Operator	10–20	Holder of an occupational diploma	30–40	8	0.15	2
4	Inspector Assistant Manager, Controller	10–20	Bachelor, Master	30–40	10	0.18	1
5	Inspector Assistant, Manager, Controller	10–20	Bachelor, Master	30–40	10	0.18	1

In this study, the Kick event and Blowout were investigated as the main scenario and outcome, respectively. Four IPLs were considered, and the fault tree related to the first two layers was drawn to calculate the probability of failure of the layers (Fig 2). The probability of failure of the third (Killing operation) and fourth (Casing and bad cementing) layers was extracted from the study of Khakzad et al. [14] equal to 0.02 and 0.025, respectively.

**Fig 3:**
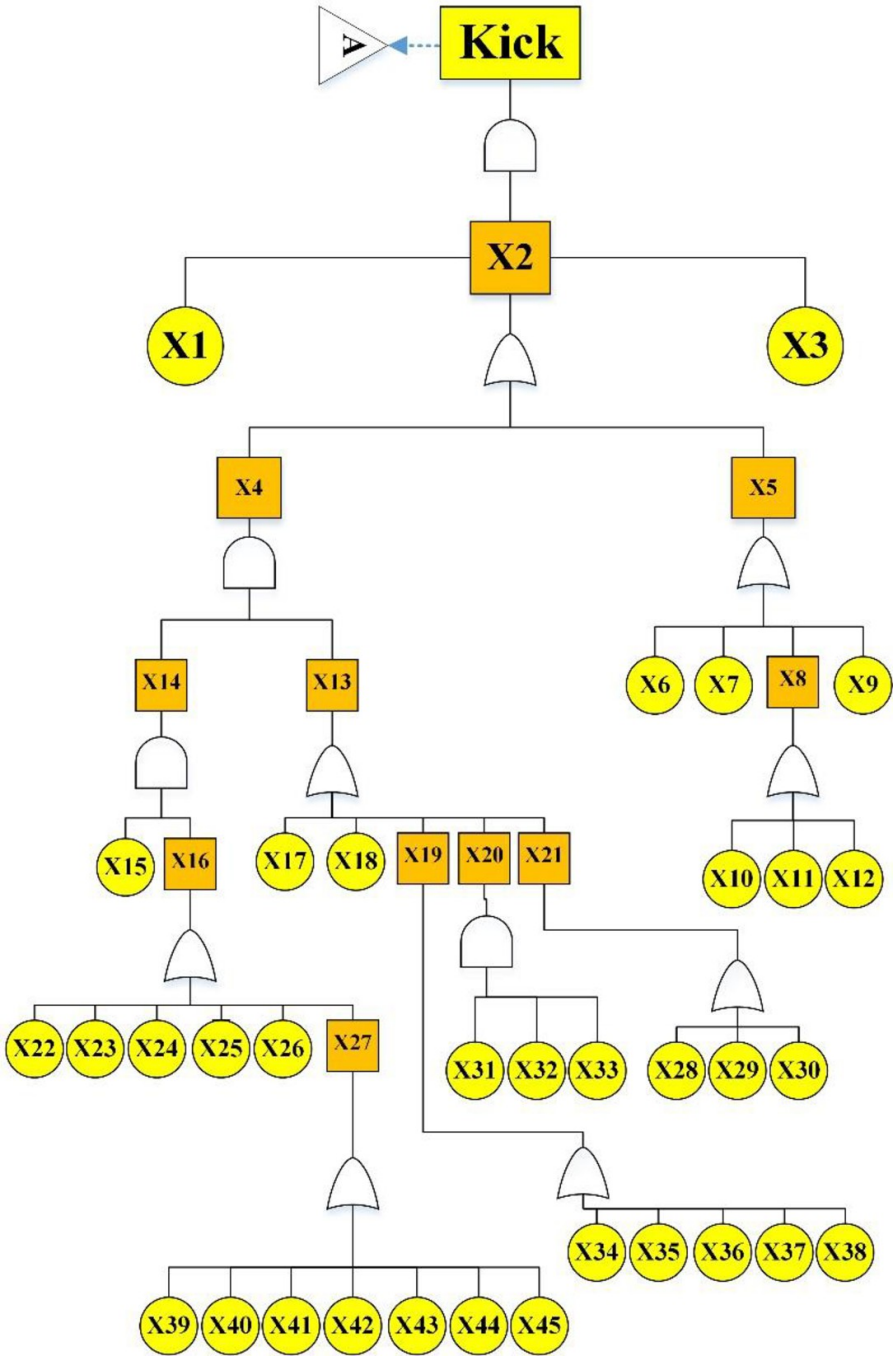
Kick FTA.

**Fig 4:**
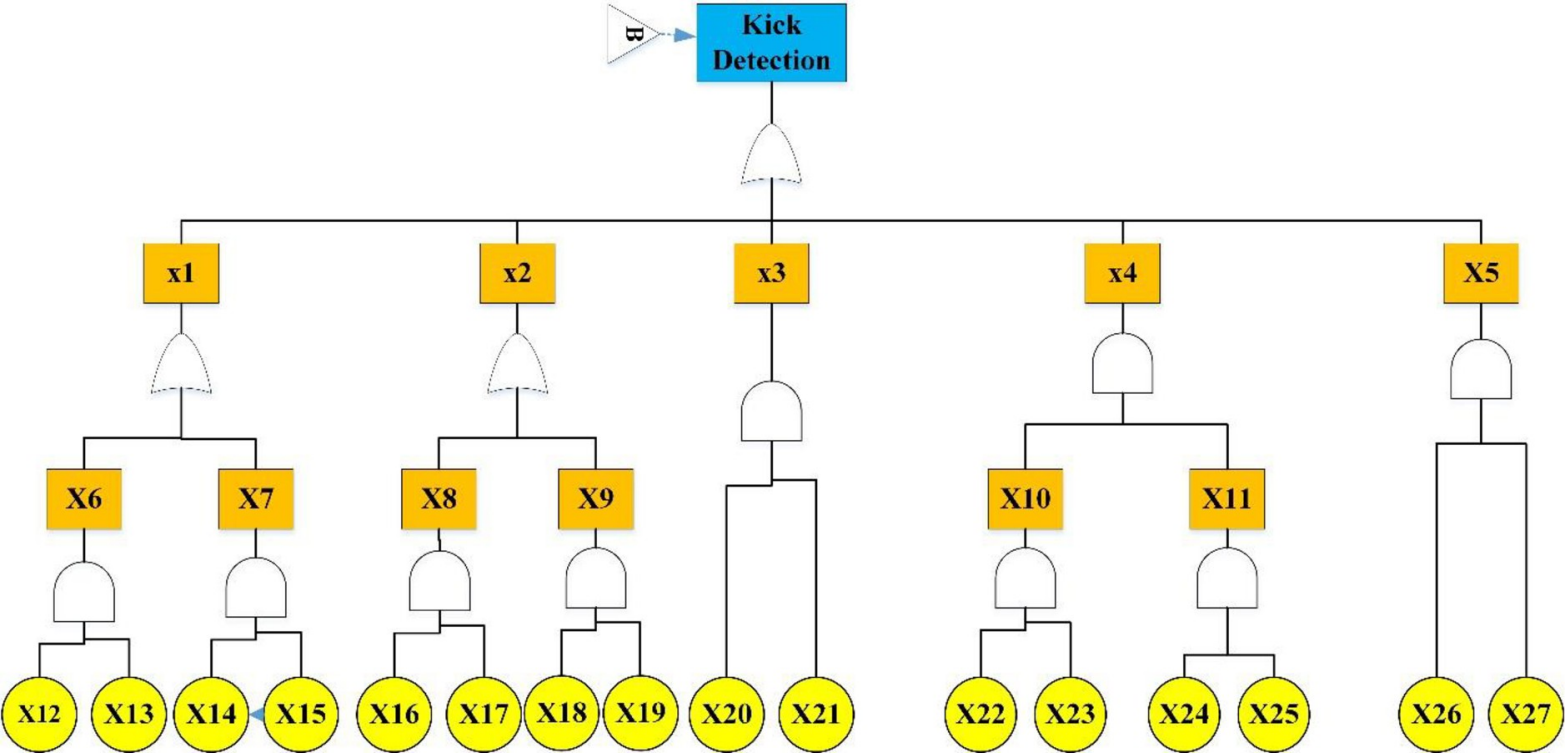
Kick detection FTA.

**Fig 5:**
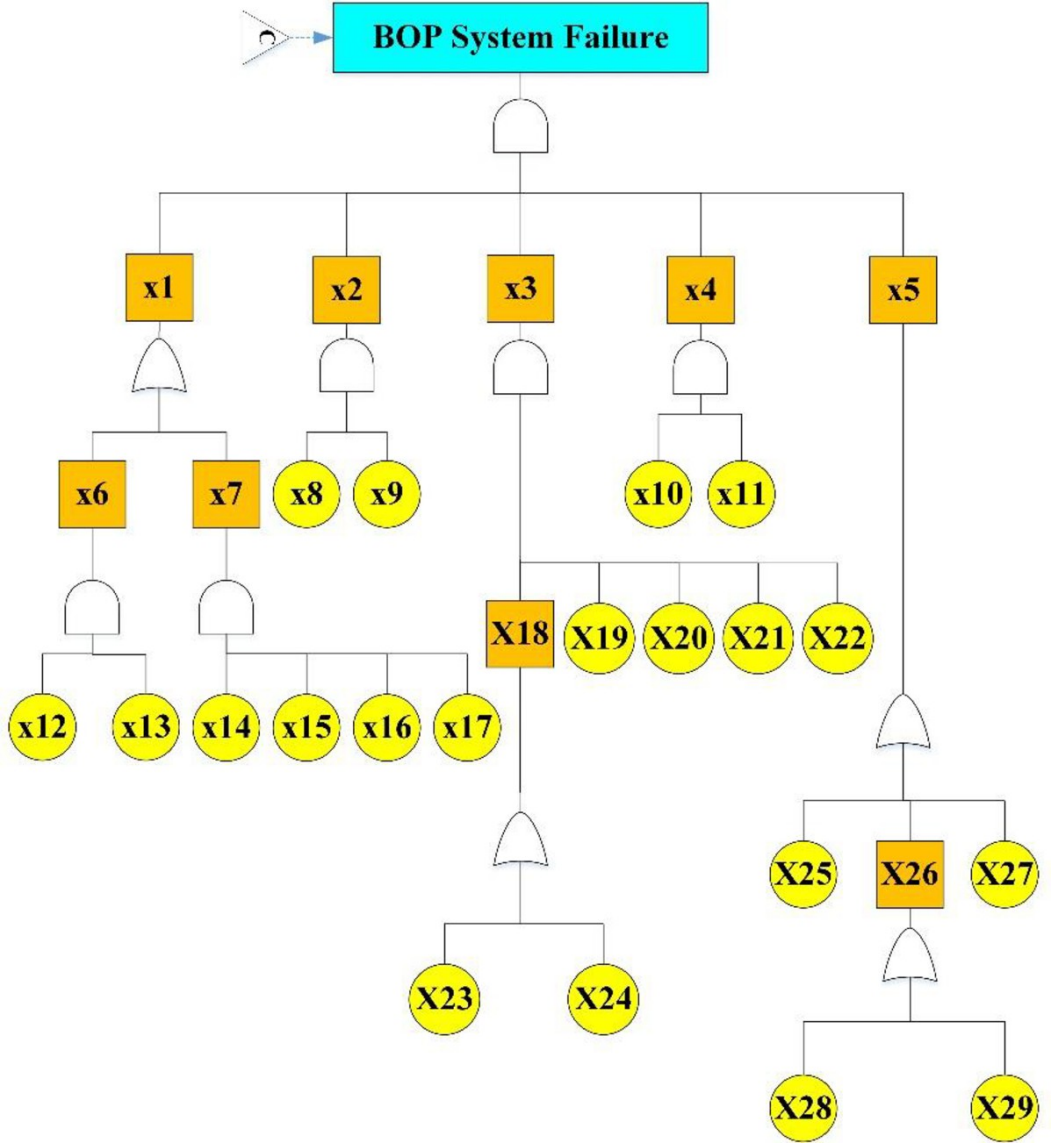
BOP system FTA.

Figs 3–5 show the fault tree related to Kick, Kick Detection, and BOP Control System respectively. In this study, fuzzy logic was used to calculate the probabilities of basic events, e.g., calculations related to the event "Pulling the pipe too fast" are explained. After receiving the opinions of experts, the relevant fuzzy numbers were combined in AFFP format (0.864, 0.6825, 0.69, and 0.528). In the next step, they were diffuzzified (0.69282). Then, the values of k (1.754572) and FP (0.017597) were obtained using the Onisawa relationship. Additionally, probabilities relating to rudimentary occurrences and intermediary events were computed, yielding outcomes that are tabulated in Table 4. As mentioned in the method, the type and position of the events in the fault tree structure, the type of gates, and the related dependencies were examined and validated with the opinions of the study experts in several sessions. Each of the events in Table 4 has a specific concept that is placed in a position in the fault tree based on the experiences of experts. For example, Mud loss or the loss of drilling mud (X16) is caused by reasons such as formation (X22), which is a geological feature of the well body. Other cases (X22 to X27) can lead to a decrease in fluid volume. Gas-cut mud (X17) is also a phenomenon that is caused by the release of gas in the mud fluid and produces bubbles that lead to a decrease in mud density (X13: Density reduction). The presence of bubbles reduces the concentration of mud.

**Table 4:** Description of events in the study.

FTA Name	Event	Description	Probability	Event	Description	Probability
Kick	X1	Efficient hydrocarbon formation	0.0055	X24	Annular losses	0.0055
	X2	Negative diffraction pressure	0.2518	X25	Bad cementing	0.005505
	X3	Sufficient permeability	0.029296	X26	Casing failure	0.005505
	X4	Low hydrostatic pressure	0.00039	X27	Surging-piston effect	0.029
	X5	Low and lost Annular Pressure Loss (APL)	2.49×10^−2^	X28	Failure in centrifuge	0.000388
	X6	Surface line failure	0.004293	X29	Failure in degasser	0.00155
	X7	Power failure	0.005505	X30	Mud cleaner equipment in adjustment	0.000699
	X8	Pump failure	0.0108	X31	Power failure	0.005505
	X9	Operator failure to notice adjustment	0.004493	X32	Agitator(mixer) failure	0.000188
	X10	Pump control failure	0.005505	X33	Settlement of mud-weight substance	0.006156
	X11	Leakage from the pump’s fluid side	0.00334	X34	Pulling the pipe too fast	0.017597
	X12	Blowing	0.001933	X35	Using Mud with high viscosity and high gel strength	0.009042
	X13	Density reduction	0.1626	X36	Having balled up a bit	0.016612
	X14	Volume reduction	0.0024	X37	Having thick wall cake	0.008513
	X15	Inadequate holes fill up	0.044593	X38	Having a small clearance between the string and the hole	0.012756
	X16	Mud loss	0.056	X39	Having and plugged drill string	0.00103
	X17	Gas-cut mud	0.0395	X40	Directing the pipes at the speed inside the well	0.004961
	X18	Abnormal pressurize	0.044593	X41	Using mud of high viscosity & and high gel strength	0.006763
	X19	Swabbing while tripping	0.063	X42	Having balled up	0.005505
	X20	Mud weight reduction	6.37×10^−09^	X43	Having Thick wall cake	0.000471
	X21	Failure in Mud treatment equipment	0.0262	X44	Having a small clearance between the string and the hole	0.009095
	X22	Formation	0.00556	X45	Using the float valve /nonreturn safety valve	0.001933
	X23	Increasing mud weight	0.005897			
Kick Detection	X1	Mud volume/ flow change	0.000856798	X15	Failure of an operator to notice the flow meter	0.0061
	X2	Circulation pressure change	5.399×10^−06^	X16	Failure of pressure gage	0.00187
	X3	Gas-cut	0.00002202	X17	Failure of an operator to notice a change in SPM	0.001987
	X4	Mud property change	2.41×10^−09^	X18	Failure of stroke meter	0.000851
	X5	Rate of Penetration (ROP) change Failure	9.95×10^−06^	X19	Failure of an operator to notice a change in P.R	0.001987
	X6	Mud tank	0.0000028	X20	Failure of gas detector	0.0003
	X7	Flow Failure	0.000854	X21	Failure of an operator to notice the gauge	0.0734
	X8	Pump Failure	3.71×10^−06^	X22	Failure of the density meter	0.0159
	X9	Pump Rate (Stroke Per Minute: SPM)	1.69×10^−06^	X23	Failure of an operator to the density meter	0.0159
	X10	Mud density	0.00025281	X24	Failure of resistivity	0.0006
	X11	Mud conductivity	0.00000954	X25	Failure of an operator to notice the conductivity change	0.0159
	X12	Failure of tank level indicator (float system)	0.04	X26	Failure of the ROP indicator	0.004590719
	X13	Failure of an operator to notice the tank level change	0.00002	X27	Failure of the ROP change	0.002167504
	X14	Failure of flow meter	0.14			
BOP Control System	X1	BOP stack failure	0.0000992	X16	Lower pipe ram failure	0.0245
	X2	Valve failure	0.000058564	X17	Blind shear ram failure	0.014
	X3	BOP control system failure	2.3820×10^−10^	X18	Power system failure	0.00572266
	X4	Line failure	0.119716	X19	4Way valve failure	0.012802685
	X5	Choke manifold failure	0.029990727	X20	Remote panel valve failure	0.014823261
	X6	Annular preventer	0.006027	X21	Signal line failure	0.01482
	X7	Ram preventer	2.058×10^−07^	X22	Accumulator line failure	0.0148
	X8	Kill valve fail	0.242	X23	Air-driven pump failure	0.002521597
	X9	Choke valve fail	0.000242	X24	Electric pump failure	0.003209155
	X10	Choke line fail	0.346	X25	Choke valve failure	0.000242
	X11	Kill line fail	0.346	X26	Hydraulic choke valve failure	0.019444405
	X12	Upper annular preventer fails	0.246	X27	Gate valve failure	0.010516
	X13	Lower annular preventer fails	0.0245	X28	Choke remote panel failure	0.009769928
	X14	Upper pipe ram fail	0.0245	X29	Hydraulic choke valve failure	0.009769928
	X15	Middle pipe ram fail	0.0245			

**Note**: FTA = fault tree analysis

It is crucial to acknowledge that X denotes various instances within three distinctive scenarios: Kick, Kick detection, and BOP systems. These sequences range from event number one until their culminating circumstances; thus, it is imperative not to conflate them.

Table 5 shows the basic and intermediate events and probabilities related to the three fault trees of the Kick, Kick Detection, and BOP Systems. According to Table 5, in the main scenario (Kick), most probabilities are related to the intermediate event X2 (Negative diffraction pressure) and X3 (Sufficient permeability). In the case of IPL1, most probabilities are related to intermediate events of X1 (Mud volume/flow change). Basic event X14 (Failure of flow meter) also has the highest probability among basic events. The most probable basic events in IPL2 are related to X10 (Choke line fail) and X11 (Kill line fail).

**Table 5:** Comparison of probabilities based on FFT and FBN.

Approach	Kick	IPL1	IPL2	IPL3	IPL4	Blowout
**FFT**	2.53×10^−2^	8.94×10^−4^	4.97×10^−21^	0.02	0.025	5.62×10^−29^
**FBN**	3.48×10^−5^	0.00089214	4.25×10^−23^	0.02	0.025	6.60×10^−34^

**Note**: IPL = Independent protection layer; FFT = Fuzzy fault tree; FBN = Fuzzy bayesian network

Ultimately, as determined by the input gate, the likelihood of failure for the Kick event amounted to 2.53×10^−2^. The failure probability of IPL1 and IPL2 was also equal to 8.94×10^−04^ and 4.97×10^−21^, respectively. Also, according to relation 9, Blow-Out probability was calculated based on the fuzzy error tree 5.62×10^−29^.

**Fig 6:**
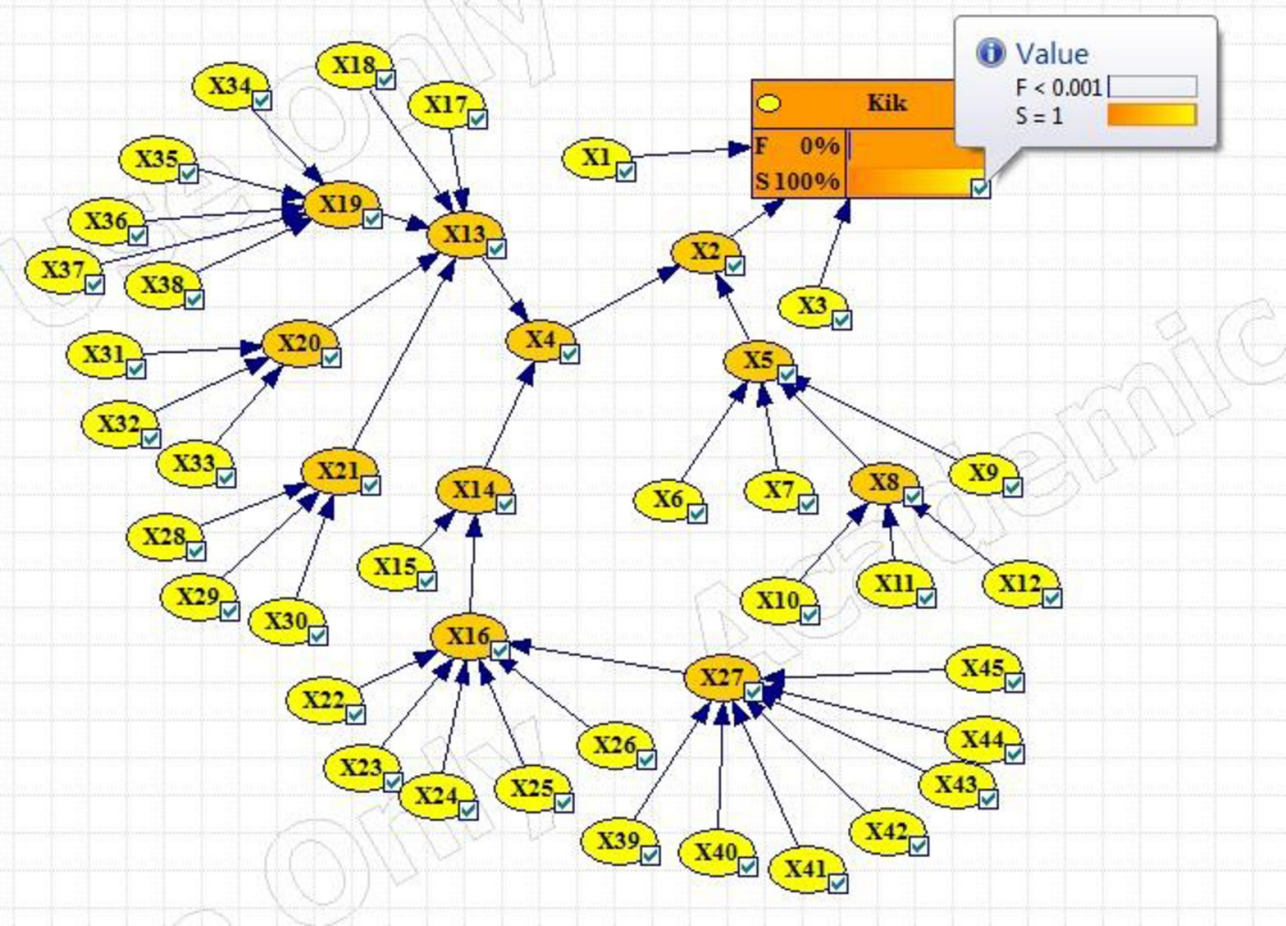
Updating the failure probabilities of the different events of Kick failure.

**Fig 7:**
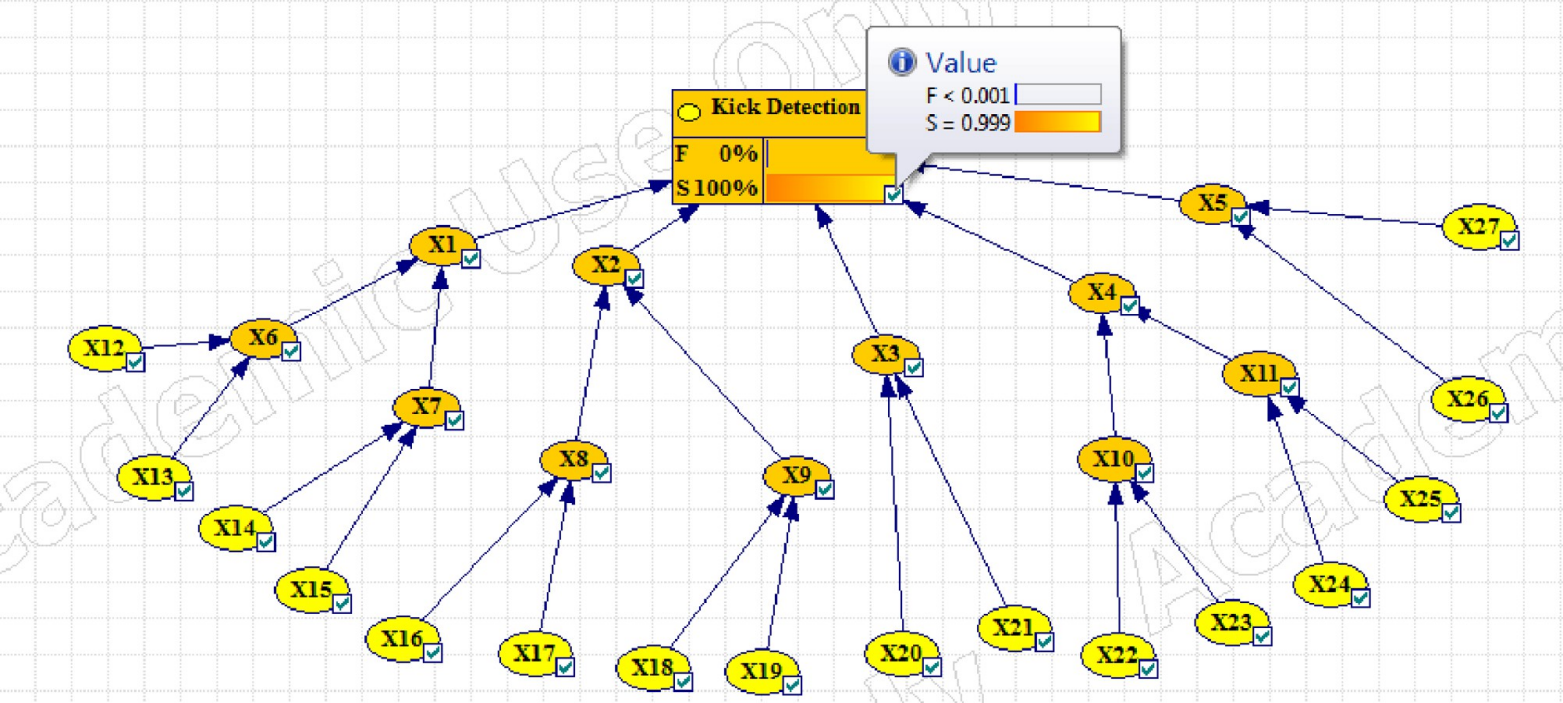
Updating the failure probabilities of the different events of Kick detection (IPL1) failure.

**Fig 8:**
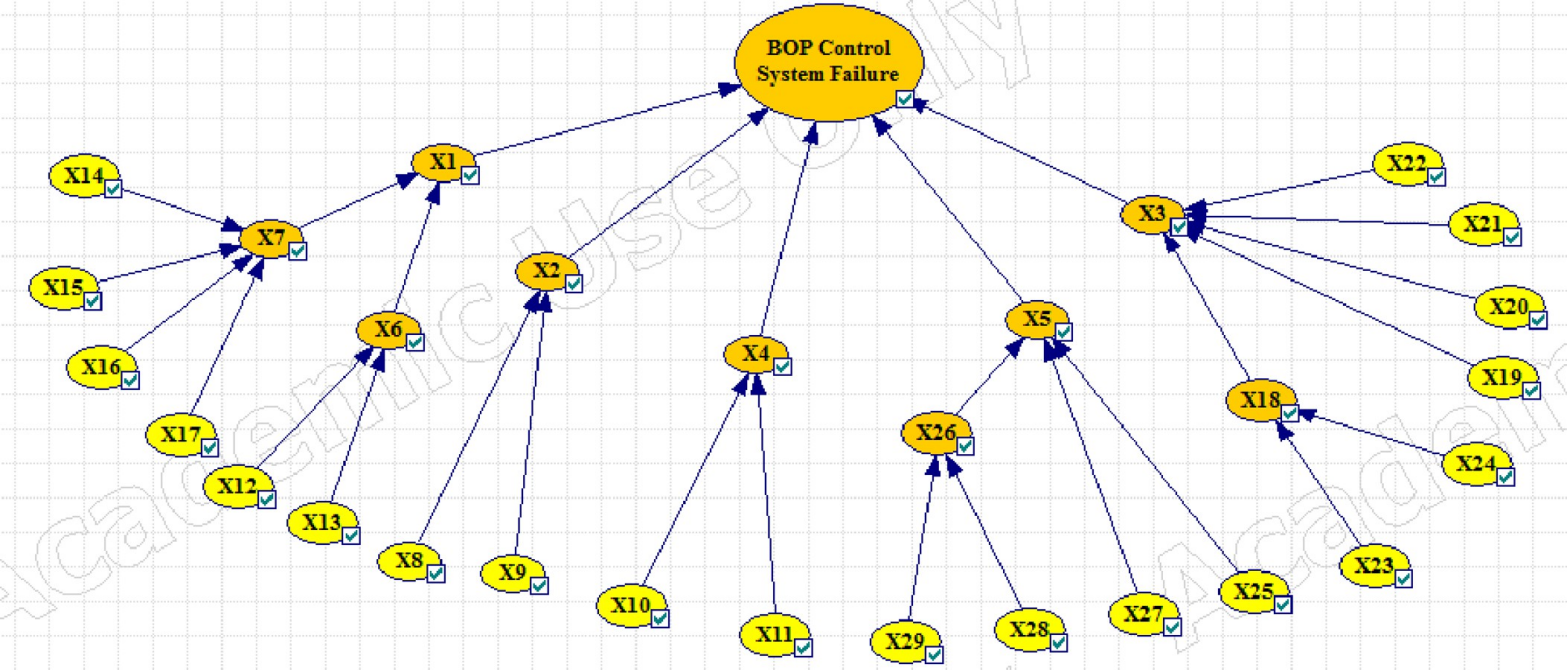
Updating the failure probabilities of the different events of BOP control system (IPL2) failure.

Then different events were transferred to the FBN in GeNIe software according to the method. Figs 6–8 respectively show the probability of failure of Kick, IPL1, and IPL2 based on a Fuzzy Bayesian Network (FBN). According to the results, the probability of failure of IPL1 and IPL2 was equal to 0.00089214469 and 4.25×10^−23^, respectively, which is very low. The probability of the Kick event was also equal to 6.60×10^−34^.

At this stage, the comparison of probabilities was done according to the results of fuzzy fault tree (FFT) and FBN, the results of which are summarized in Table 5.

**Fig 9:**
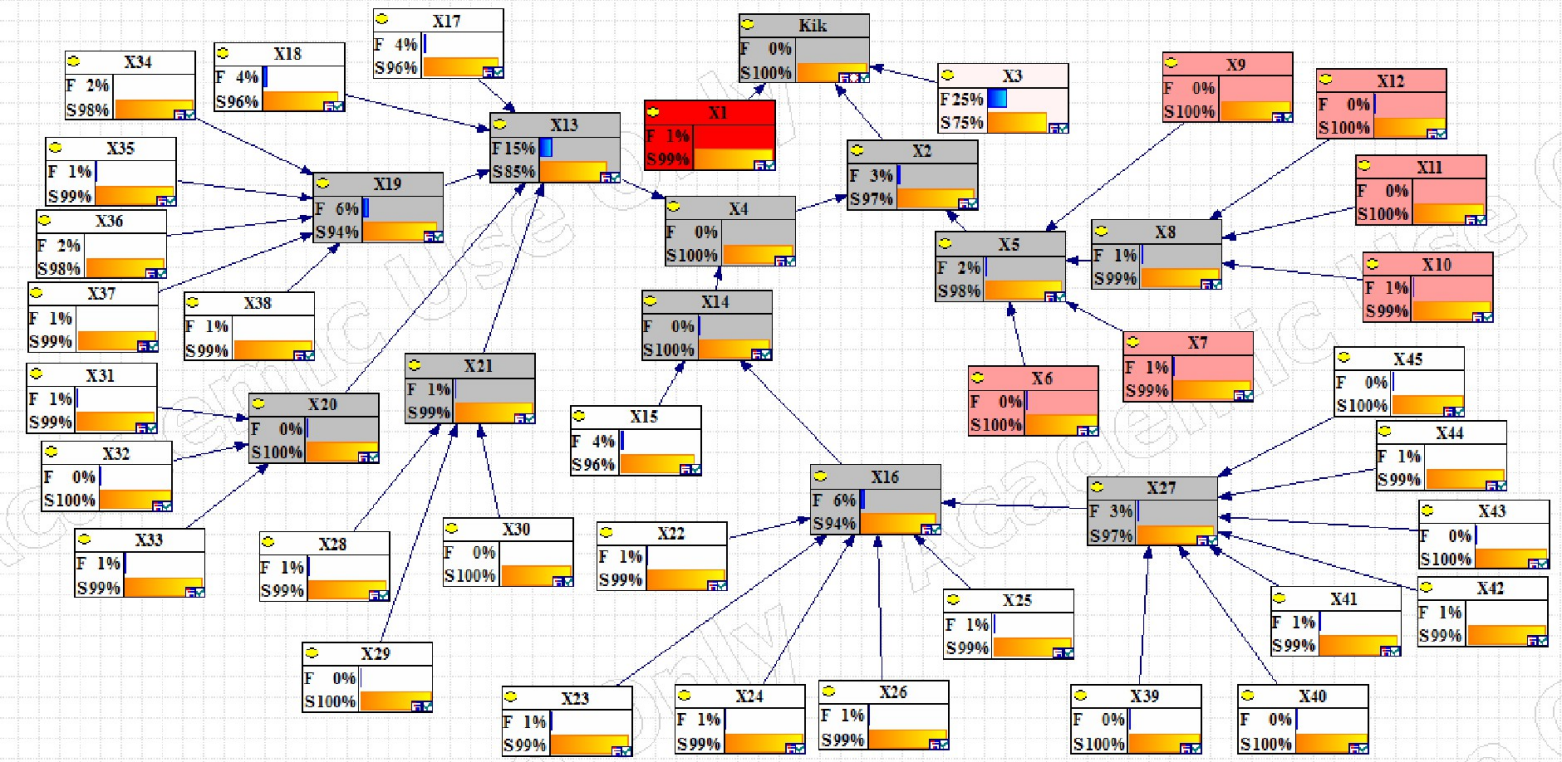
Sensitivity analysis of Kick.

**Fig 10:**
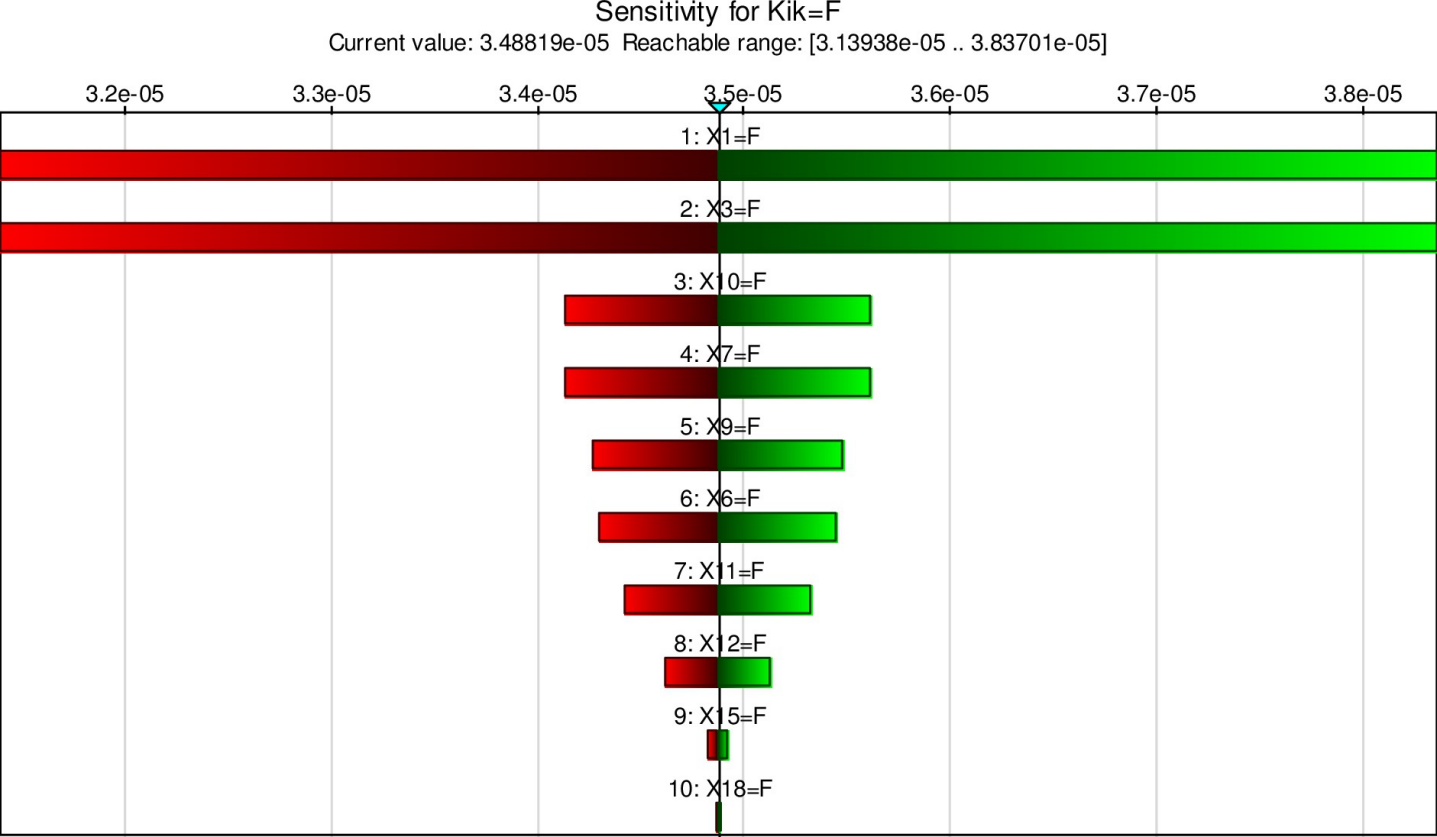
Critical basic events of Kick.

Sensitivity analysis was performed in GeNIe software to determine the critical events of Kick. Figs 9 and 10 show the failure probabilities of different events by color. According to the figure, the most critical events are X1. Events X3, X10, X7, X9, and X6 are other important basic events.

## 4. Discussion

Comprehensive risk management requires an all-encompassing approach to risk assessment. Therefore, in this study, the research employed a combined approach based on the FBN. The complexity of the dynamic mechanism of the blowout, risk factors specific to oil wells and special conditions, the complexity of decisions to properly respond to well control events, operational and environmental uncertainties, and the weakening of barriers due to time factors, require the improvement of new risk assessment techniques to consider risk factors [48, 49]. In addition, one of the innovations of the present study was to consider multiple barriers after the Kick event. Also, the probability of failure of IPL1 and IPL2 was calculated using separate fault trees.

The lack of a data bank for various failures, numerous cultural and social differences, and different characteristics and types of equipment always make the systems face uncertainty [17]. Using fuzzy logic to calculate the failure rate of Kick and different IPLs can help reduce uncertainty. Feng et al’s recommendation advised relying on past databases or expert opinions to assess gas pipeline failures [50].

In this study, the risk of Kick, based on FFT and FBN, was equal to 2.53×10^−2^ and 3.48×10^−5^, respectively. Blowout probability was equal to 5.62×10^−29^ and 6.60×10^−34^ respectively. Several barriers were considered to prevent blowouts in this study. Therefore, the probability of Blowout was estimated to be very low because many barriers played an effective role in reducing the probability. In a study, Aliabadi et al analyzed the root causes of blowouts in oil and gas wells in the drilling industry. Kick was identified as the main event, and the possibility of the consequences of the blowout was investigated using Bow-Tie’s technique and FBN [4]. The present study’s BN model boasted various capabilities [16, 51], resulting in reduced uncertainty and enabling complex causal relationships and successive dependent failures to be modeled. This renders FBN results more realistic compared to FFT outcomes. In this study, the selection of BN was done with two goals. The first was to fix the shortcomings of the Bow-Tie model and the second was to reduce the uncertainty in the conditions of lack of data.

Throughout this analysis, several layers or control barriers were meticulously considered after contemplating potential kick events or main scenarios. Additionally, fault trees corresponding with these first two layers were also developed. A sequential modeling approach was used in the SHIPP method and the study of Pouyakian et al. In these studies, not only process factors, but also human and organizational factors and barriers were used [17, 52, 53]. Thus, in the present study, the barriers were placed in a logical approach and sequence to minimize the failure rate and, finally, the probability of a blowout.

In a study, Igbani et al. conducted an oil well blowout risk assessment with HAZOP and Bow-Tie [54]. Shafiee et al used the combined FMEA and FTA model in a study to assess the risk of Subsea Blowout Preventers equipment [55]. Minimum cuts were determined using the FTA method and weighted based on Birnbaum’s measure. These minimum weighted cuts were evaluated based on the FMEA method, and their risk priority number (RPN) was obtained [55].

In a review study that Ebrahim conducted to investigate the practical application of the Bow-Tie approach in the gas and oil industry, it was emphasized that the Bow-Tie method is graphical, clearly showing the threats and consequences, and creating a better understanding than other methods. The biggest disadvantage of this method is uncertainty in quantification [56]. In a study, Khakzad used the Bow-Tie method and BN for QRA of drilling activities. They stated that the dynamic nature of blowout events requires that the rapid changes of physical parameters and time-dependent failure of barriers be considered during the lifetime of a well [57].

The Bow-Tie method is widely used in the safety analysis, identification, and risk assessment of complex systems. On the other hand, this method has many defects that are very important in process industries. Static structure, lack of flexibility to combine new knowledge and evidence, lack of updating the probabilities of basic events, lack of ability to reduce data uncertainty and use rare information, and the inability to consider the conditional dependence of the basic events are among the shortcomings of the Bow-Tie method [14, 22, 58] that should be fixed. Through the combination of the FBN with quantitative or qualitative risk assessment techniques, a higher level of precision and effectiveness can be achieved in process safety assessment studies while mitigating uncertainties [44, 59]. Currently, the existing approaches in risk assessment should consider this capability due to the dynamics of variables affecting process accidents. BN has a flexible and adaptable feature for dynamic modeling and analysis of a wide range of scenarios. It can consider conditional dependence between events with common causes. Additionally, this method has the capability of inductive and deductive reasoning, making the network structure dynamic and allowing for the updating of the probability of root events. This results in the built model being closer to reality and reducing uncertainty [14, 60, 61]. In a study, Yin et al used BN for blowout analysis. This method covers the complex features of geological conditions, as well as surface and subsea BOP failures. BOP failure was one of the main causes of the blowout in the study [62]. Cai et al. also used BN to evaluate the reliability of the subsea blowout preventer control system. They investigated the failure rate of different components and systems in terms of reliability using BN [26]. In another study, Cai et al. investigated the application of DBNs to quantitatively assess the risk of human factors on offshore blowouts. The structure of human factors in a marine blowout was shown using a pseudo-fault tree (PFT), and a method for translating PFT to BN and DBN, considering repair, was proposed [27].

Therefore, to address the limitations of the model created by the Bow-Tie method, this model inputted into the FBN and became a suitable causal model for the accident scenario. FBNs quantitatively model the intensity of communication between variables, and with access to new information, the conditional belief about them is automatically updated [21, 58].

Within the framework of the FBN, probabilities for main events are derived from CPTs, which are computed based on conditional probabilities linking intermediate nodes to their corresponding dependent root nodes. It is worth mentioning that certain assumptions must be made to facilitate the conversion algorithm from FTA to BN. These assumptions are as follows: a) events are binary (work and failure) b) events are statistically independent c) The relationships between events and causes are expressed by logic gates, and d) The root of the FTA is an undesired event to be analyzed [46]. Therefore, the most critical events were identified using FBN capabilities. The results showed that events X1, X3, X10, X7, X9, and X6 are the most important critical events. The likelihood of a failure in the principal event is significantly diminished by excising each of these events. Continuous health and safety training [19], standard operating procedures (SOP) ensuring their periodic update with system changes, and implementation of management systems [63] can significantly reduce the severity of consequences along with risk analysis methods. The presence of ambiguity when establishing the odds of IPL3 and IPL4 barriers succumbing to failure, coupled with oversight regarding perils brought forth by Mother Nature like deluges, seismic activity, thunderbolts, and whatnot, were identified as the limitations of the present study.

## 5. Conclusion

### 5.1 The proposed approach

In this study, a comprehensive approach based on FBN was presented to reduce the uncertainties of oil well blowout. This innovative approach aims to generate comprehensive insights into this complex phenomenon while minimizing inherent ambiguity. The use of BN makes the model more realistic and reduces uncertainty due to the dynamic evaluation of various variables and the static nature of the Bow-Tie structure.

The absence of a data bank for various failures, many social and cultural differences, and exclusive features and types of equipment always make the systems face uncertainty. Applying fuzzy logic in calculating the failure rate and estimating conditional probabilities can help reduce uncertainty. Thus, in this study, it can be said that with the mentioned multiple features, deductive and inductive reasoning in BN, and fuzzy logic capabilities, a more realistic model for blowout evaluation was presented. Therefore, FBN showed more realistic results than FFT. It delves into the realm of inductive reasoning, employing its profound capabilities to ascertain the likelihood of events and their subsequent consequences. Moreover, employing deductive reasoning fosters a dynamic network structure that facilitates the possibility of data updates. Therefore, the presented approach can be used in many process industries, especially in the prevention of kicks and subsequent blowouts.

### 5.2 Conclusion of the case study

The results of this study can be used in both a preventive and reactive approach. In this study, by removing each of the critical events, it is possible to achieve favorable results in this field. In this approach, in addition to examining the probability of kick failure, the probability of barrier failure was also considered. Also, with this approach, the adequacy of safety layers can be examined in two preventive and reactive modes and the weak points of the controls can be identified. In this method, the adequacy of safety layers to reduce possible consequences (e.g. blowout) by identifying critical events and prioritizing them was checked, and if necessary, new layers or controls should be defined by decision-makers.

The findings unveiled through the sensitivity analysis remarkably indicate that X1 holds paramount significance in oil well blowouts. With an unwavering focus on continuous safety and health training alongside honing pertinent skills, compliance with Standard Operating Procedures (SOPs), and ensuring their periodic update congruent with system alterations, this comprehensive approach promises to substantially diminish adversities within this study’s purview. Therefore, it is necessary to provide these comprehensive approaches to reduce the costs and risks of drilling and lead to the development of pressure drilling techniques to eliminate inconsistencies.
